# Peripheral Artery Tonometry Reveals Impaired Endothelial Function before Percutaneous Coronary Intervention in Patients with Periprocedural Myocardial Injury

**DOI:** 10.1155/2021/5598120

**Published:** 2021-07-15

**Authors:** Zhangwei Chen, You Zhou, Jiasheng Yin, Qinglai Gao, Ao Chen, Yan Xia, Danbo Lu, Dong Huang, Juying Qian, Junbo Ge

**Affiliations:** ^1^Department of Cardiology, Zhongshan Hospital, Fudan University, Shanghai Institute of Cardiovascular Diseases, National Clinical Research Center for Interventional Medicine, Shanghai 200032, China; ^2^Department of Cardiology, Zhongshan Hospital, Fudan University, Shanghai Geriatric Medical Center, Shanghai Institute of Cardiovascular Diseases, National Clinical Research Center for Interventional Medicine, Shanghai 200032, China; ^3^Department of Cardiology, People's Hospital of Wulian, Rizhao, Shangdong Province 262300, China

## Abstract

**Background:**

Periprocedural myocardial injury (PMI) is a most common complication of percutaneous coronary intervention (PCI). Microembolization and inflammation underlying PMI could lead to coronary microvascular dysfunction (CMD) and vice versa. Reactive hyperemia index (RHI) assessed by peripheral artery tonometry (PAT) has been considered as a noninvasive method to assess endothelial function and CMD, which could be useful to predict PMI.

**Methods:**

268 patients suspected with stable coronary artery disease (CAD) and scheduled for elective coronary angiography were enrolled. RHI was measured by using the Endo-PAT2000™ device before angiography. The association among RHI, PMI, and cardiovascular events was further assessed.

**Results:**

In this cohort, 189 patients (70.5%) were diagnosed with CAD and 119 patients (44.4%) underwent drug-eluting stent (DES) implantation. Compared with patients without CAD, CAD patients had lower RHI (1.88 ± 0.55 vs. 2.02 ± 0.58, *P* < 0.05). Patients with PMI had a lower RHI before angiography (1.75 ± 0.37 vs. 1.95 ± 0.50, *P* < 0.05). Receiver operating characteristic curve analysis of RHI revealed an area under the curve (AUC) of 0.61, with a sensitivity of 62.7% and specificity of 50.0% to predict PMI. Moreover, we found that CAD patients with RHI ≤ 1.81 had a higher incidence of composite cardiac events after stenting (adjusted hazard ratio (HR) 3.31, 95% confidence interval (CI) 1.07–10.22, *P* < 0.05).

**Conclusions:**

RHI assessment through PAT could be a promising method to predict PMI before the procedure. RHI is associated with increased risk of long-term adverse cardiac events after DES implantation.

## 1. Introduction

PCI has been a standard approach to achieve revascularization of obstructive coronary CAD. The improvement of techniques and equipment dramatically decreases the incidence of severe complications after PCI, such as stent thrombosis, perforation, and death [1]. However, PMI emerges as a frequent complication of PCI with an incidence of 5–70% depending on the diagnostic criteria [2–4]. Previous studies imply an association between PMI and adverse cardiovascular events [4–6]. The diagnosis of PMI depends on the values of cardiac troponin (cTn) or creatine kinase-MB (CK-MB) after the procedure. Yet, no ideal method has been found to predict or prevent PMI effectively. Herein, we intend to find out risk factors or examinations that could identify patients at high risk of PMI before the procedure, so that we can adopt preventive measures to reduce the damage of PMI.

The most common mechanisms of PMI could be classified into two types: one is due to side branch occlusion proximal to the target lesion of PCI, and the other is due to microvascular dysfunction distal of the treated lesion [7]. The distal type is believed to be the dominant type of PMI, which is closely related with embolization of the arteriole, vascular dysregulation, and microcirculatory dysfunction [8–11]. CMD is a crucial pathophysiologic damage caused by PMI, which, in turn, could be an important risk factor that predisposes patients to PMI. PAT, which detects the reactive hyperemia-induced changes in digital peripheral pulse amplitude to evaluate peripheral endothelial function [12], was not only closely related with cardiovascular risk factors [13] but also considered as a valuable noninvasive way to evaluate coronary microvascular function [14, 15]. Hence, PAT could be a useful method to identify patients at high risk of PMI before the procedure.

Impaired endothelial function detected by PAT was associated with higher adverse cardiac event rate during follow-up [16]. Nevertheless, the cardiovascular prognosis of CAD is not only associated with endothelial function and traditional risk factors but also determined by its severity and further treatment strategies, such as optimal medication and coronary revascularization. There were few studies reporting the association between RHI and long-term cardiovascular events after DES implantation. Komura et al. found that lower RHI was correlated with angiographic in-stent restenosis (ISR) [17]. However, angiographic ISR could not represent the cardiac events completely. Therefore, we designed this prospective study to confirm the value of PAT in discriminating patients at risk of PMI as well as predicting long-term adverse cardiovascular events after DES implantation.

## 2. Methods

### 2.1. Ethics Statement

This study was approved by the Ethical Committee of Zhongshan Hospital, Fudan University (Approval No: B2016-018, Date: 2016/02/29). All patients provided their written, informed consent. The study was carried out in accordance with the principles of the Declaration of Helsinki.

### 2.2. Patient Recruitment

From May 2015 to November 2015, 268 patients suspected with stable CAD admitted for elective PCI were enrolled. Exclusion criteria were acute coronary syndrome, malignant hypertension, New York Heart Association (NYHA) class III to IV heart failure, cardiomyopathy, and cardiac valvular disease. Patients with active infection, carcinoma, immunological disorders, and liver or kidney dysfunction (eGFR < 60 mL/min/1.73 m^2^ or liver enzyme >3 × upper reference limit (URL)) were also excluded.

### 2.3. Measurement of RHI

RHI was measured with Endo-PAT2000™ (Itamar Medical, Israel) before coronary angiography by cardiologists who were blinded to the results of clinical characteristics and laboratory testing. This measurement was arranged in a quiet test room at 8 am–10 am with temperature set to 24–28 centigrade. All vasoactive medications (such as calcium antagonists and nitrate) were discontinued at least 24 h prior to testing. According to previous studies and device protocols, a blood pressure cuff was placed on one upper arm, while the other arm served as a control. PAT probes were placed on each index finger for continuous recording of the pulse signal. After a 10 min equilibration period, the blood pressure cuff was inflated to suprasystolic pressure (200 mmHg or 60 mmHg plus systolic blood pressure) for 5 min. Then, the cuff was deflated to induce reactive hyperemia and PAT was recorded for a further 5 min. The pulse amplitude recordings were automatically analyzed and quantified as RHI.

### 2.4. Clinical Records and Laboratory Measurements

CAD was diagnosed when at least one lesion led to a >50% reduction in lumen diameter in coronary angiography. Coronary angiography, syntax score evaluation, and intervention were performed by cardiologists blinded to laboratory testing and RHI. Venous serum samples were collected on admission and 16–20 hours after PCI for laboratory measurements.

### 2.5. Endpoints and Clinical Long-Term Follow-Up

PMI was defined as a cTnT value above the 99% URL after the procedure. Patients were followed up with a median 18-month interval through telephone consultation or outpatient clinic attendance. The primary outcomes were major adverse cardiovascular events (MACEs), including cardiac death, nonfatal myocardial infarction (MI), stroke, target vessel revascularization (TVR), and rehospitalization driven by heart failure.

### 2.6. Statistical Analysis

All statistical analyses were performed with SPSS for Windows, release 25.0 (IBM SPSS, Inc., Chicago, IL, USA). Continuous variables are presented as mean ± standard deviation (SD) or median with the interquartile range (IQR). Categorical data are expressed as counts and percentages. Chi-square or Fisher's exact test was used to compare the frequency for categorical variables. Means for continuous variables were compared by Student's t or Mann–Whitney U test. Logistic regression was performed to identify risk factors and the Spearman test for correlation analyses. Kaplan–Meier survival analysis was conducted to compare the difference in incidence of MACEs. The prognostic impact of RHI was assessed with a univariable and multivariable Cox proportional hazard model, adjusted to age, male, hypertension, diabetes, smoking, low-density lipoprotein cholesterol, and hemoglobin. All *P* values were two sided, and *P* < 0.05 was considered statistically significance. Figures were plotted using GraphPad Prism, version 9.0 (GraphPad Software, San Diego, CA, USA).

## 3. Results

### 3.1. Study Population

268 patients suspected with stable CAD undergoing elective coronary angiography were recruited in this study. 189 patients (70.5%) were diagnosed with CAD by angiography, and 119 patients (44.4%) accepted DES implantation. When PMI was defined as a post-PCI cTnT value > 99% URL, 51 out of the 119 patients (42.9%) had PMI.

### 3.2. Population Characteristics and RHI

**Figure 1:**
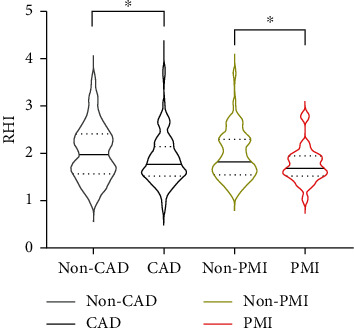
Violin plot of RHI in different groups. Distribution of RHI in different patient groups was presented in a violin plot. The width of the box represents the density of values. Solid lines and dotted lines represent the median and interquartile range of RHI, respectively. Patients with CAD or PMI had lower RHI compared with patients without CAD or PMI, separately. ^*∗*^*P* < 0.05. CAD, coronary artery disease; PMI, periprocedural myocardial injury; and RHI, reactive hyperemia index.

Compared with patients without CAD, CAD patients had a lower RHI (1.88 ± 0.55 vs. 2.02 ± 0.58, *P* < 0.05) (Figure 1). The cohort was divided into two groups according to the median value of RHI (1.81). No difference in age, gender, blood pressure, body mass index (BMI), and history of diabetes or smoking was found (Table 1). Only triglyceride was detected higher in patients with RHI ≤ 1.81 (1.94 ± 1.71 vs. 1.59 ± 0.95, *P* < 0.05). Concomitant medications that could interfere with RHI were similar between the two groups. Nitrates, *β*-receptor blockades, and calcium channel blockades were withheld for at least 24 h before PAT measurement. Despite similarities in risk factors of CAD, patients with RHI ≤ 1.81 had a higher syntax score (17.3 ± 8.3 vs. 12.0 ± 6.6, *P* < 0.001). RHI was negatively correlated with syntax score (Spearman *r* = −0.345, *P* < 0.001) as well as hs-CRP (Spearman *r* = −0.162, *P* < 0.05). 60 (45.8%) and 79 (57.7%) patients underwent stenting in the high RHI and low RHI groups, respectively. The incidence of PMI was 37% in patients with RHI > 1.81 compared with 47.7% in patients with RHI ≤ 1.81, which, however, was not statistically significant yet.

### 3.3. Baseline and Procedural Characteristics of PMI

As shown in Table 2, patients with PMI had a lower RHI (1.75 ± 0.37 vs. 1.95 ± 0.50, *P* < 0.05) and higher syntax score (17.4 ± 8.4 vs. 13.4 ± 6.3, *P* < 0.01) compared with patients without PMI. Besides, patients with PMI tended to have higher LDL, higher total cholesterol, and higher HDL. Neither demographic characteristics, including age, gender, hypertension, diabetes, smoking, and BMI, nor clinical examinations, including creatinine, hs-CRP, lipoprotein (a), ejection fraction (EF), and left ventricular diameter, were significantly different. Patients with PMI had more stents implanted and, therefore, longer stent length (52.8 ± 29.8 vs. 38.0 ± 21.5 mm, *P* < 0.01). Antiplatelet therapy and use of statins, as well as bailout use of glycoprotein IIb\IIIa inhibitors, were similar between the two groups. No dissection, branch loss, perforation, or cardiac tamponade was witnessed (data not shown) in either group.

### 3.4. Risk Factors of PMI

Binary logistic analysis was performed to evaluate the role of RHI on the occurrence of PMI. Our results demonstrated that RHI (odds ratio (OR) 0.35, 95% confidence interval (CI) 0.4–0.86, *P* < 0.05) was a protective factor while stent length (OR 1.02, 95%CI 1.01–1.04, *P* < 0.01) was a risk factor of PMI (Table 3). Stent length could only be determined during the procedure, which made RHI a valuable indicator in the early prediction and prevention of PMI. Furthermore, we constructed a ROC curve to assess the ability of RHI to predict the occurrence of PMI. The area under the curve to predict PMI was 0.61 (95% CI: 0.51–0.71; *P* < 0.05) (Figure 3). An RHI cutoff value of 1.83 had a sensitivity of 62.7% and specificity of 50.0% to detect PMI.

### 3.5. RHI and Long-Term Outcome

**Figure 2:**
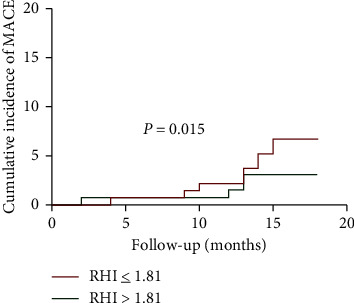
Cumulative incidence of MACEs. Patients with RHI ≤ 1.81 had a higher risk of MACEs (*P* < 0.05). MACEs, major adverse cardiovascular events; RHI, reactive hyperemia index.

**Figure 3:**
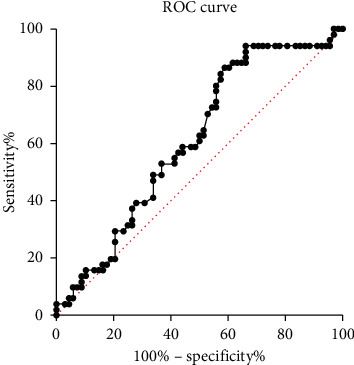
ROC curve for the prediction of PMI. The dotted diagonal line is the null hypothesis with AUC = 0.50. RHI < 1.83 had a sensitivity of 62.7% and specificity of 50.0% to predict PMI with AUC = 0.61 (95% CI 0.51–0.71). AUC, area under the curve; CI, confidence interval; PMI, periprocedural myocardial injury; RHI, reactive hyperemia index; ROC, receiver operating characteristic.

Patients were followed up for a mean period of 18 months, during which 20 patients had adverse events (Table 4). No patients died. One patient had myocardial infarction (MI). 13 patients underwent target vessel revascularization. Four patients were hospitalized for deterioration of heart failure. Ischemic stroke was diagnosed in 2 patients. Kaplan–Meier survival analysis was performed to compare the difference in the cumulative incidence of MACEs between patients with high and low levels of RHI. Using the median value of RHI (1.81), we found that patients with low RHI were more likely to suffer from MACEs (log-rank *P* < 0.05) (Figure 2). The impact of RHI on the risk of MACEs was also evaluated by univariate and multivariate Cox regression analyses. RHI ≤ 1.81 increased the risk of MACEs in CAD patients (HR 3.34, 95%CI 1.10–10.16; *P* < 0.05), even after adjustment to age, gender, hypertension, diabetes, smoking, hemoglobin, and LDL (adHR 3.31, 95%CI 1.07–10.22, *P* < 0.05).

### 3.6. Discussion

In the past decades, the development of both equipment and technique has made PCI a mainstay in the treatment of obstructive coronary disease. While severe complications of PCI keep declining over the years, there has not been an ideal way to predict PMI and cut down the incidence. Coronary microvascular dysfunction plays an essential role in PMI, which is not only the consequence of microcirculatory damage but also a crucial risk factor to exacerbate myocardial injury. Assessment of CMD relies on functional assessment of microcirculation, which can be performed invasively, such as index of microvascular resistance (IMR) [18, 19], and noninvasively, including positron emission tomography (PET) and cardiac magnetic resonance (MRI) [20, 21]. However, these methods are limited by availability, cost, or exposure to radiation. RHI assessment by PAT has been validated as a noninvasive way to evaluate peripheral and coronary microvascular endothelial function [12, 14, 15]. Herein, we designed this prospective study to determine if preprocedural RHI is a risk factor and predictor of PMI. Patients with comorbidities that would influence cTnT values were excluded, such as ACS, renal disease, and autoimmune disease. In order to exclude the influence of environment factors, we conducted PAT in a solitary room at a stipulated time with constant illumination, temperature, and noise control. Medications that could interfere with PAT were withheld, and patients were inculcated to avoid stress since the night before measurement. RHI was calculated automatically by using the recorder to prevent the objective error of the operator.

In this cohort, the incidence of PMI was consistent with our previous study [22]. Compared with the patients without CAD, CAD patients had lower RHI. RHI was also negatively associated with syntax score and hs-CRP. These indicated that RHI was associated with the severity of CAD and, more importantly, the inflammatory state. This dose makes sense since previous studies have confirmed the inflammatory state is interrelated with atherosclerosis and endothelial dysfunction [19, 23, 24]. Logistic regression implied RHI was a protective factor of PMI, and ROC analysis indicated RHI could predict the occurrence of PMI with an AUC of 0.61. Among risk factors that could be identified before PCI, including hypertension, diabetes, smoking, dyslipidemia, and BMI, RHI is the only indicator that correlated with PMI. Stent length was another risk factor of PMI; however, it could not be determined before the angiography was performed, which limited its use in the early prediction of PMI. Although the sensitivity and specificity of RHI require validation in larger populations, PAT proves to be a promising noninvasive method to predict PMI early and conveniently. The median value of RHI was 1.81 in our study, which was different from previous reports [25, 26]. Variance in the level of RHI may be associated with different inclusion criteria, races, age distributions, and diseases of interest. Larger-scale studies are needed to determine a uniform cutoff value of RHI for risk identification.

Moreover, RHI is reported to be closely related with risk factors and early stage of CAD as well as poor cardiovascular prognosis [13, 14, 16, 27]. In this study, we did not find the association between RHI and traditional risk factors, such as diabetes, hypertension, and BMI. Probably, it is because the sample size was relatively small. Besides, these patients recruited had less comorbidities and relatively short course of disease, in which case the influence of risk factors has not posed an impact strong enough on RHI. DES has become one of the most important treatments in CAD; nevertheless, few studies reported the impact of RHI on long-term outcome after DES implantation. We found that the median level of RHI (1.81) was associated with a higher incidence of MACEs, even adjusted by traditional risk factors. It has been argued that PMI as well as PMI-related poor prognosis could be a result of plaque burden or procedural complexity. In the present study, we also noticed RHI was adversely associated with syntax score, which reflected the status of atherosclerosis. However, as we mentioned in the previous paper [22], PMI posed a greater impact on patients with low risk of adverse cardiovascular events, which could not be explained by either coronary lesions or the procedure. Therefore, endothelial and microcirculatory dysfunction could be a crucial cause of poor prognosis, which was not included in syntax score but an appropriate target of PAT. Besides, we have built multivariate predictive models of MACEs based on traditional risk factors with and without RHI. Preliminary results indicated inclusion of PAT improved C-statistic and decreased the Akaike information criterion even in the presence of syntax score; that is, RHI proved to be an important predictor of MACEs holding additional value. Due to the relatively small number of patients, these results were tentative and not displayed yet. Moreover, PAT is a convenient method to be conducted, which can be a valuable way to track the fluctuation of RHI in order to assess the effectiveness of therapy and guiding clinical practice in the future.

There are some limitations in our study. First, this is a single-centered study with a relatively small sample size. However, the present study has been a relatively large one to investigate the independent role of RHI on the long-term composite outcome after DES implantation. Second, this study was conducted on a rather homogenous population. Patients with comorbidities such as ACS and renal or liver dysfunction were excluded. As a result, additional research is needed to verify if these findings could be extended to other cohorts. Third, we could not acquire the details about RHI when MACEs happened, for RHI measured at follow-up may reflect the simultaneous state of endothelial function and more closely related with the type of MACEs. However, this will not affect the conclusion that baseline RHI was a predictor of PMI and correlated with high risk of MACEs. Fourth, multicentered research is pended to bring out a uniform cutoff value of RHI to predict, and the specificity and sensitivity of RHI need to be tested in larger populations.

## 4. Conclusions

RHI assessed by PAT could be a promising predictor of PMI before the procedure. Low RHI is correlated with high risk of long-term MACEs in CAD patients after DES implantation.

## Figures and Tables

**Table 1:** Demographics and clinical characteristics.

	Total (268)	RHI > 1.81 (131)	RHI ≤ 1.81 (137)	*P*
Age	62.9 ± 9.0	63.2 ± 8.8	62.6 ± 9.3	0.654
Male	185 (69.0)	87 (66.4)	98 (71.5)	0.365
Hypertension	170 (63.4)	80 (61.1)	90 (65.7)	0.432
Diabetes	60 (22.3)	28 (21.4)	32 (23.4)	0.697
Smoking history	95 (35.4)	42 (32.1)	53 (38.7)	0.257
BMI	24.7 ± 3.0	24.5 ± 3.2	24.9 ± 2.8	0.228
CAD	189 (70.5)	87 (66.4)	102 (74.5)	0.149
Syntax score	14.9 ± 8.0	12.0 ± 6.6	17.3 ± 8.3	<0.001
Coronary slow flow^*∗*^	11 (4.1)	4 (3.1)	7 (5.1)	0.396

*Laboratory and auxiliary examinations*				
Systolic blood pressure (mmHg)	132.4 ± 12.3	131.6 ± 12.2	133.2 ± 12.4	0.592
Diastolic blood pressure (mmHg)	80.0 ± 8.2	79.0 ± 8.4	81.0 ± 8.0	0.100
NT-proBNP (pg/ml)	214.2 ± 536.7	215.1 ± 523.9	213.0 ± 550.6	0.461
Creatinine (mg/dl)	78.2 ± 16.9	76.8 ± 17.3	79.5 ± 16.6	0.187
CK (U/L)	94.4 ± 47.6	94.0 ± 50.8	94.8 ± 44.4	0.317
CK-MB (U/L)	12.9 ± 8.2	13.0 ± 10.2	12.7 ± 5.2	0.941
hs-CRP (mg/L)	3.94 ± 7.27	3.55 ± 7.17	4.35 ± 7.40	0.096
Hemoglobin (g/L)	134.4 ± 14.5	132.9 ± 13.9	135.9 ± 14.8	0.091
Platelet (×10^9/L)	207.5 ± 64.2	205.9 ± 62.1	209.0 ± 66.4	0.827
Total cholesterol (mmol/L)	3.82 ± 0.97	3.73 ± 0.86	3.91 ± 1.07	0.252
Low-density lipoprotein (mmol/L)	1.97 ± 0.81	1.94 ± 0.74	1.99 ± 0.87	0.892
Triglyceride (mmol/L)	1.77 ± 1.40	1.59 ± 0.96	1.94 ± 1.71	0.038
High-density lipoprotein (mmol/L)	1.14 ± 0.46	1.15 ± 0.57	1.13 ± 0.33	0.938
Lp (a) (mmol/L)	349.6 ± 485.8	351.8 ± 468.7	347.5 ± 503.5	0.370
HbA1c (%)	6.2 ± 1.4	6.1 ± 1.2	6.3 ± 1.5	0.111

*Echocardiography*				
LA (mm)	38.9 ± 4.3	38.1 ± 4.4	39.5 ± 0.4	0.010
LVEDD (mm)	47.1 ± 4.5	46.5 ± 4.2	47.6 ± 4.8	0.050
LVESD (mm)	30.3 ± 4.3	30.0 ± 3.7	30.8 ± 4.8	0.090
SPAP (mmHg)	32.4 ± 6.3	32.7 ± 7.0	32.1 ± 5.7	0.784
EF (%)	64.4 ± 7.0	64.3 ± 7.3	64.5 ± 6.6	0.906

*Medication*				
Statin	237 (88.4)	113 (86.3)	124 (90.5)	0.277
Β-blockade	175 (65.2)	86 (65.6)	89 (65.0)	0.906
Nitrates	132 (49.3)	59 (45.0)	73 (53.3)	0.177
Calcium channel blockade	68 (25.4)	35 (26.7)	33 (24.1)	0.621

Data are shown as mean ± SD or *n* (%). ^*∗*^Slow flow was defined as a reduced thrombolysis in a myocardial infarction (TIMI) flow grade of 2 or lower in coronary angiography. BMI, body mass index; CAD, coronary artery disease; hs-CRP, hypersensitive C-reactive protein; EF, ejection fraction; LA, left atrium; LVEDD, left ventricular end-diastolic diameter; LVESD, left ventricular end-systolic diameter; Lp (a), lipoprotein (a); PMI, periprocedural myocardial injury; SPAP, systolic pulmonary arterial pressure.

**Table 2:** Baseline and procedural characteristics of PMI.

	Total (119)	Non-PMI (68)	PMI (51)	*P*
Age	63.7 ± 9.5	64.0 ± 9.8	63.2 ± 9.3	0.476
Male	91 (76.4)	50 (73.5)	41 (80.4)	0.382
Hypertension	81 (68.1)	44 (64.7)	37 (72.5)	0.364
Diabetes	25 (21.0)	16 (23.5)	9 (17.5)	0.687
Smoking history	49 (41.1)	25 (36.8)	24 (47.1)	0.259
BMI	24.8 ± 3.2	24.7 ± 3.2	24.9 ± 3.3	0.573
Syntax score	15.2 ± 7.5	13.4 ± 6.3	17.4 ± 8.4	0.008
RHI	1.86 ± 0.46	1.95 ± 0.50	1.75 ± 0.37	0.039

*Laboratory and auxiliary examinations*				
NT-proBNP (pg/ml)	225.1 ± 582.9	229.7 ± 735.7	219.2 ± 298.2	0.006
Creatinine (mg/dl)	78.3 ± 15.8	76.6 ± 15.0	80.6 ± 16.6	0.088
CK (U/L)	94.6 ± 45.6	96.0 ± 50.8	92.9 ± 38.3	0.658
CK-MB (U/L)	12.8 ± 3.8	12.8 ± 4.3	12.8 ± 3.1	0.596
hs-CRP (mg/L)	5.07 ± 9.41	3.95 ± 6.08	6.46 ± 12.3	0.691
Hemoglobin (g/L)	136.2 ± 14.1	135.4 ± 13.8	137.3 ± 14.7	0.512
Platelet (×10^9/L)	208.3 ± 68.2	205.4 ± 76.4	212.3 ± 56.0	0.262
Total cholesterol (mmol/L)	3.86 ± 1.02	3.70 ± 0.99	4.08 ± 1.03	0.032
Low-density lipoprotein (mmol/L)	2.04 ± 0.83	1.88 ± 0.77	2.24 ± 0.86	0.024
Triglyceride (mmol/L)	1.83 ± 1.70	1.97 ± 2.10	1.66 ± 0.92	0.912
High-density lipoprotein (mmol/L)	1.08 ± 0.30	1.06 ± 0.34	1.12 ± 0.24	0.025
Lp (a) (mmol/L)	339.2 ± 438.2	300.0 ± 298.6	391.8 ± 485.2	0.393
HbA1c (%)	6.1 ± 1.2	6.1 ± 1.4	6.1 ± 0.9	0.400

*Echocardiography*				
LA (mm)	39.2 ± 4.2	39.2 ± 4.5	39.4 ± 3.8	0.900
LVEDD (mm)	46.7 ± 4.0	46.5 ± 4.5	47.2 ± 3.2	0.350
LVESD (mm)	30.2 ± 3.4	30.1 ± 3.6	30.4 ± 3.1	0.644
SPAP (mmHg)	32.9 ± 7.4	33.1 ± 8.2	32.6 ± 6.3	0.973
EF (%)	64.7 ± 6.1	65.0 ± 6.2	64.5 ± 6.2	0.529

*Medication*				
Aspirin	115 (96.6)	64 (94.1)	51 (100)	0.134
Statin	118 (99.2)	67 (98.5)	51 (100)	1.000
Β-blockade	87 (73.1)	54 (79.4)	34 (66.7)	0.117
Nitrates	74 (62.2)	39 (57.4)	35 (68.6)	0.060
Calcium channel blockade	29 (24.3)	16 (23.5)	13 (25.5)	0.805

*PCI*				
Stent length (mm)	44.4 ± 26.3	38.0 ± 21.5	52.8 ± 29.8	0.002
Use of GBI	20 (16.8)	11 (16.2)	9 (17.6)	0.832
Average stent diameter (mm)	3.06 ± 0.44	3.11 ± 0.45	2.99 ± 0.41	0.149

*Target vessel number*				
1	96 (80.7)	58 (85.3)	38 (74.5)	0.140
2	20 (16.8)	9 (13.2)	11 (21.6)	0.229
3	3 (2.5)	1 (1.5)	2 (1.7)	0.576

Data are shown as mean ± SD or *n* (%). ^*∗*^Slow flow was defined as a reduced thrombolysis in a myocardial infarction (TIMI) flow grade of 2 or lower in coronary angiography. BMI, body mass index; hs-CRP, hypersensitive C-reactive protein; GPI, glycoprotein IIb/IIIa receptor inhibitor; EF, ejection fraction; LA, left atrium; LVEDD, left ventricular end-diastolic diameter; LVESD, left ventricular end-systolic diameter; Lp (a), lipoprotein (a); PCI, percutaneous coronary intervention; SPAP, systolic pulmonary arterial pressure.

**Table 3:** Risk factors of PMI.

	Odds ratio (95% CI)	*P*
Age ≥65 y	0.67 (0.33–1.40)	0.291
Male	1.48 (0.61–3.55)	0.384
Hypertension	1.44 (0.65–3.18)	0.365
Diabetes	0.70 (0.28–1.73)	0.437
Dyslipidemia	1.47 (0.64–3.38)	0.362
Smoking history	1.53 (0.73–3.20)	0.260
Body mass index	1.02 (0.91–1.14)	0.738
Stent length	1.02 (1.01–1.04)	0.004
Target vessel number	1.82 (0.83–4.00)	0.137
Average stent diameter	0.52 (0.22–1.24)	0.141
RHI	0.35 (0.14–0.86)	0.022

RHI, reactive hyperemia index.

**Table 4:** Long-term outcomes and RHI.

.	RHI ≤ 1.81	RHI > 1.81	HR	*P*	adHR	*P*
MACEs^*∗*^	16	4	3.34 (1.10–10.16)	0.033	3.31 (1.07–10.22)	0.038
Nonfatal myocardial infarction	1	0	—	0.784	—	—
Target vessel revascularization	10	3	—	0.193	—	—
Cardiac death	0	0	—	1	—	—
Rehospitalization driven by heart failure	4	0	—	0.784	—	—
Ischemic stroke	1	1	—	0.053	—	—

Values are given as *n*. Adjustments were made for age (>65 y), gender, hypertension, diabetes, smoking, hemoglobin, and LDL. Only data with *P* < 0.05 were shown. ^*∗*^MACEs were a composite of cardiac death, nonfatal myocardial infarction, ischemic stroke, target vessel revascularization, and rehospitalization driven by heart failure.

## Data Availability

Readers can access the data supporting the conclusions of this study by contacting the correspondence author.
